# Comment on “Therapeutic plasma exchange in acute liver failure: Unravelling its efficacy and impact”

**DOI:** 10.1016/j.htct.2026.106514

**Published:** 2026-08-11

**Authors:** Akshaya Tomar

**Affiliations:** Department of Immunohematology & Blood Transfusion, Armed Forces Medical College, Pune, India

To the editor:

I read with interest the article entitled “Therapeutic plasma exchange in acute liver failure: Unravelling its efficacy and impact” by Sonal Sonu et al., published in *Hematology, Transfusion and Cell Therapy* (2026;48(3):106268) [1].The authors are to be commended for addressing the critical role of therapeutic plasma exchange (TPE) in acute liver failure (ALF), a condition with high mortality, where donor scarcity limits transplantation.

However, several methodological and interpretative flaws warrant careful consideration to contextualize the findings appropriately.

First, the lack of a control group significantly limits causal inference. This retrospective single-arm study reports improvements in bilirubin, transaminases, and a Model for End-Stage Liver Disease (MELD) scores after TPE, but without a concurrent control group receiving standard medical therapy alone, these changes cannot be definitively attributed to the intervention. Prior randomized controlled trials, such as that by Larsen et al., have employed control arms to establish efficacy, and their absence here weakens causal claims [2].

Second, the study is underpowered and lacks adjustment for multiple comparisons. With only 25 patients, the authors perform 11 separate laboratory comparisons and post-hoc subgroup analyses across three outcome groups (mortality, discharged, and discharged against medical advice (DAMA) patients without applying corrections such as Bonferroni. The reported p-value for indirect bilirubin (p = 0.008) and the non-significant trend for platelets (p = 0.063) would not withstand such adjustment. This increases the risk of type I errors and overinterpretation of marginal findings.

Third, there is a discordance between biochemical improvement and patient outcomes. While discharged patients showed a substantial reduction in MELD scores (33.8-11.9), non-survivors had no significant change (36.7-28.9; p = 0.080). The authors interpret this as TPE improving prognosis in survivors, but an equally plausible explanation is selection bias, as survivors might have been predisposed to clinical improvement. Without a control group, one cannot distinguish treatment effect from natural history. Additionally, the DAMA patient group showed significant reductions in MELD scores yet left due to financial constraints; this intriguing finding is understudied without a long-term follow-up.

Fourth, key clinical outcomes are missing. The study does not report transplantation rates, 30- or 90-day mortality, or time to encephalopathy resolution, despite citing these as rationales for TPE. The authors acknowledge that transplant services were unavailable at their institution yet conclude TPE serves as a “bridge to transplantation” without providing data on how many patients were successfully bridged [3]. The hospital stay varied substantially (8.9 days for non-survivors vs. 56.0 days for survivors), suggesting significant confounding by illness severity rather than a direct treatment effect.

Fifth, the safety analysis lacks clarity regarding the denominator. Adverse events are reported as percentages (itching 15%, urticaria 14%, hypocalcemia 20%), but it is unclear whether these represent per-patient or per-procedure rates. With a median of 3–5 sessions per patient, event rates per procedure would be substantially lower. Standardized apheresis complication grading, as recommended by Mokrzycki et al., would improve comparability with other series [4].

I offer the following constructive suggestions. First, perform a propensity-matched or historical control analysis using ALF patients managed conservatively during the same period. Second, include subgroup analyses stratified by etiology or baseline MELD score to address the limited interpretability of treatment effects across heterogeneous populations. Although not explicitly required by the American Society for Apheresis guidelines, such analyses align with broader methodological expectations outlined in the STROBE statement, which emphasizes transparent evaluation of factors that may influence outcomes [5]. Third, report clinically meaningful endpoints including transplantation-free survival at fixed time points (e.g., 28 days) and time to encephalopathy resolution. Fourth, apply appropriate statistical corrections for multiple comparisons or present the analysis as hypothesis-generating. Finally, provide follow-up data on DAMA patients to assess durability of TPE effects.

I believe these points warrant clarification to better interpret the study’s contribution. Despite the limitations, the work highlights the feasibility and safety of TPE in ALF in a resource-limited setting, and I encourage the authors to extend their analysis with a controlled design.

## Funding statement

This research did not receive any specific grant from funding agencies in the public, commercial, or not-for-profit sectors.

## Ethical consent

Not applicable.

## Conflicts of interest

None.
